# Study on Calcified Alkali Leaching of Vanadium-Extracted Tailings and Preparation of Barium Orthovanadate

**DOI:** 10.3390/nano15241889

**Published:** 2025-12-17

**Authors:** Jinwei Qu, Yiqiu Wang, Xinyu Hao, Na Ma

**Affiliations:** 1 Guangxi Key Laboratory of Calcium Carbonate Resources Comprehensive Utilization, Hezhou University, Hezhou 542899, China; qujinwei2008@163.com (J.Q.); wyq15225979273@163.com (Y.W.); 2 State Environmental Protection Key Laboratory of Mineral Metallurgical Resources Utilization and Pollution Control, Wuhan University of Science and Technology, Wuhan 430081, China

**Keywords:** vanadium-extracted tailings, calcified alkaline leaching, leaching rate, vanadium precipitation, barium orthovanadate

## Abstract

While vanadium-extracted tailings contain valuable components, their utilization is difficult due to their high sodium content. In this work, a new oxygen-pressure calcification and alkaline leaching strategy to achieve barium orthovanadate vanadium precipitation is developed to realize the resourceful recycling and utilization of vanadium-extracted tailings. First, the preparation of barium orthovanadate via calcified alkaline leaching and vanadium precipitation was studied, and the effects of CaO addition, NaOH concentration, leaching temperature, and liquid–solid ratio on the leaching rates of sodium and vanadium were evaluated in single-factor experiments. Under the optimum leaching conditions (CaO addition of 20%, alkali concentration of 150 g·L^−1^, leaching temperature of 180 °C, and liquid–solid ratio of 10:1), the leaching rates of vanadium and sodium reached 85.25% and 82.36%, respectively. Subsequently, the vanadium-containing leaching solution was subjected to a vanadium precipitation test, and the effects of pH, Ba(OH)_2_ addition (expressed as $n_{\mathrm{Ba}}/n_{V}$), vanadium precipitation temperature, and vanadium precipitation time on the vanadium precipitation rate were investigated. Under the optimum vanadium precipitation conditions (pH 14, $n_{\mathrm{Ba}}/n_{V}$ = 1.5:1, temperature of 30 °C, reaction time of 60 min), a vanadium precipitation rate of more than 99% was achieved. The precipitated vanadium product of this reaction was confirmed to be Ba_3_(VO_4_)_2_ with a purity of more than 99%. Notably, the wastewater generated during the test process can be mixed with an alkali and returned to the leaching process for reuse, and the dealkalized residue can be used as a raw material for ore reduction in iron smelting processes.

## 1. Introduction

Vanadium is a rare metal with critical roles in steel manufacturing, materials science, chemical manufacturing, aerospace, and other industries [1]. In nature, vanadium is primarily found in vanadium–titanium magnetite, lignite vanadium ore, potassium–vanadium–uranium mica ore, and petroleum-associated ore deposits [2]. China possesses abundant vanadium resources, which are widely distributed across the country in the form of vanadium-rich shale and vanadium–titanium magnetite deposits. Of these, the deposits in Sichuan and Hebei provinces account for 80% of the overall vanadium market. According to the 2021 Chinese Mineral Resources Census, the total vanadium–titanium magnetite reserves in China reached 951.2 million tons [3].

The beneficiation and smelting of vanadium–titanium magnetite produces raw materials (vanadium slag) that are suitable for vanadium extraction. The residue obtained after vanadium slag undergoes sodium calcination and water leaching is typically referred to as vanadium-extracted tailings. These tailings are rich in valuable metals such as manganese, chromium, titanium, and vanadium. Moreover, due to their iron content (generally greater than 40%), vanadium-extracted tailings are a common blast furnace feedstock for iron smelting. However, these blast furnaces are prone to caking because the vanadium-extracted tailings also have an alkali content of approximately 5% (on a Na_2_O basis), which negatively impacts the blast furnace service life and limits the utilization of these tailings [4].

For every ton of vanadium pentoxide produced from vanadium–titanium magnetite, up to 10 tons of vanadium-extracted tailings are generated. Against the backdrop of current societal development, China produces approximately 600,000 tons of vanadium-extracted tailings each year, with Sichuan Pangang and Hebei Chenggang responsible for about 80% of this total. In addition to occupying a significant amount of land, these vanadium-extracted tailings also contribute to environmental pollution [5]. Furthermore, the hexavalent chromium in vanadium-extracted tailings poses a significant threat to human health. In recent years, numerous domestic and international studies have been conducted on the secondary utilization of vanadium-extracted tailings as a resource, including the production of low-vanadium iron [6], the preparation of vanadium flux [7], the extraction of secondary vanadium via sodium calcination [8], the use of direct pressure acid leaching for vanadium extraction [9], and vanadium recovery via extraction [10]. However, these technologies generally have low vanadium recovery efficiencies and are costly in terms of energy or acid consumption. Consequently, these technologies do not have sufficient processing capacities, and they are not suitable for large-scale, low-cost industrial applications [11,12,13]. In the context of global advocacy for ‘green development’, the question of how to utilize vanadium-extracted tailings in a clean, efficient, and high-value manner has become an urgent issue of significant scientific and practical value. Therefore, developing economically viable and environmentally friendly strategies for treating vanadium-extracted tailings would be of great significance.

In this study, an oxygen-pressure calcified alkali leaching technology is developed to remove the alkali and extract the vanadium from vanadium-extracted tailings. The treated residue has a Na_2_O content of less than 0.5%, fully meeting the requirements for ore blending in blast furnace ironmaking. Existing vanadium precipitation processes for treating the vanadium-containing leaching solution (such as the acidic ammonium salt method, the ammonium metavanadate method, the calcium vanadate method, the sodium vanadate method, and the iron vanadate method) have many shortcomings, including long reaction times and the high consumption of reagents and energy. Therefore, this study proposes a novel and efficient barium orthovanadate precipitation method for the recovery of vanadium in the leaching solution. Notably, a high vanadium precipitation rate exceeding 99% is achieved within just 30 min, and the final Ba_3_VO_4_ product purity is greater than 99%.

## 2. Experiment

### 2.1. Experimental Raw Material

**Figure 1:**
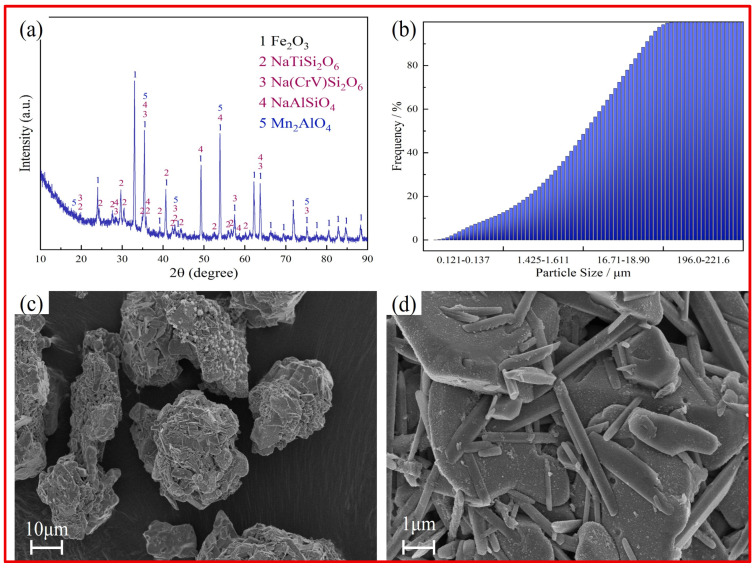
Characterization of vanadium-extracted tailings (**a**) XRD pattern (**b**) particle size (**c**,**d**) SEM images.

**Figure 2:**
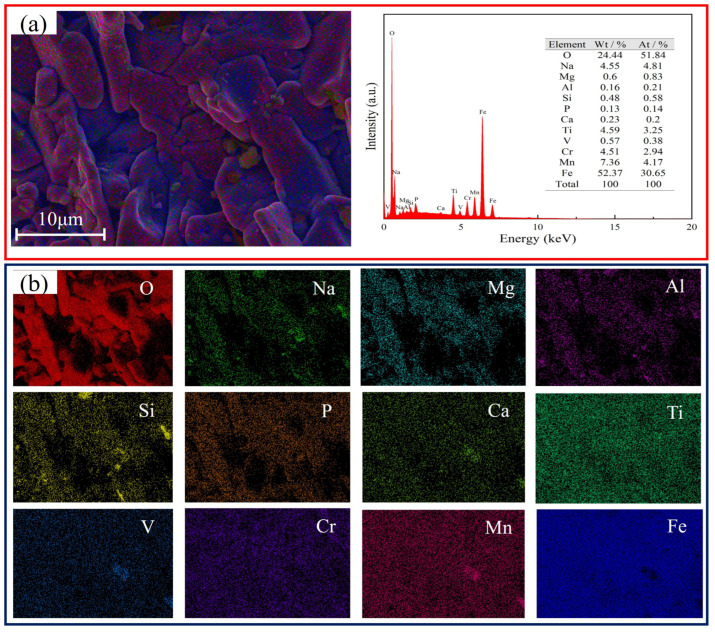
SEM-EDS mappings of vanadium-extracted tailings. (**a**) Elemental analysis results; (**b**) mappings.

The vanadium-extracted tailings used in this work were supplied by the Pangang Group (Panzhihua, China). These tailings were sieved to a particle size of less than 75 µm prior to use. Table 1 shows the main chemical components of the tailings, which primarily contain Fe, Si, Ti, Mn, Na, and V. The vanadium content of the tailings is much higher than that of vanadium–titanium magnetite. Figure 1a shows the X-ray diffraction (XRD) pattern of the vanadium-extracted tailings. The main identified phases are hematite (Fe_2_O_3_), titanium-containing sodium silicate (NaTiSi_2_O_6_), and aluminosilicate (NaAlSiO_4_). Additionally, the vanadium primarily exists in the form of pyroxene (Na(CrV)Si_2_O_6_). It should be noted that the vanadium-containing phases in vanadium-extracted tailings are difficult to fully leach [14,15,16,17]. The particle size analysis of the vanadium-extracted tailings is displayed in Figure 1b, showing an average particle size of approximately 20 µm. According to the scanning electron microscopy (SEM) images in Figure 1d, the tailings mostly exhibit lump and rod-like morphologies. Energy-dispersive X-ray spectroscopy (EDS) analysis (Figure 2a) shows that the vanadium-extracted tailings contain a variety of elements, with iron (Fe) accounting for a relatively high proportion alongside significant amounts of sodium (Na). Elemental mapping images reveal an uneven distribution of elements such as Fe, Cr, Mn, Ti, V, and Na, indicating the complex composition of the vanadium-extracted tailings.

**Table 1:** Main chemical components of vanadium extracted tailings (mass fraction, %).

Fe_2_O_3_	SiO_2_	TiO_2_	MnO	Na_2_O	Al_2_O_3_	Cr_2_O_3_	MgO	CaO	V_2_O_5_
40.76	21.82	9.72	7.38	5.77	5.35	3.51	2.02	1.76	1.91

### 2.2. Experimental Equipment and Instruments

The vanadium-extracted tailings were screened using stainless-steel standard sieves, leaching tests were conducted using stainless-steel pressure reactors, and vanadium precipitation tests were carried out using a constant-temperature water bath under stirring. The phase composition, microstructure, and elemental composition of the vanadium tailings, leaching residues, and barium orthovanadate were analyzed using XRD, SEM/EDS, X-ray photoelectron spectroscopy (XPS), X-ray fluorescence (XRF), and inductively coupled plasma mass spectrometry (ICP-MS).

### 2.3. Experimental Process

**Figure 3:**
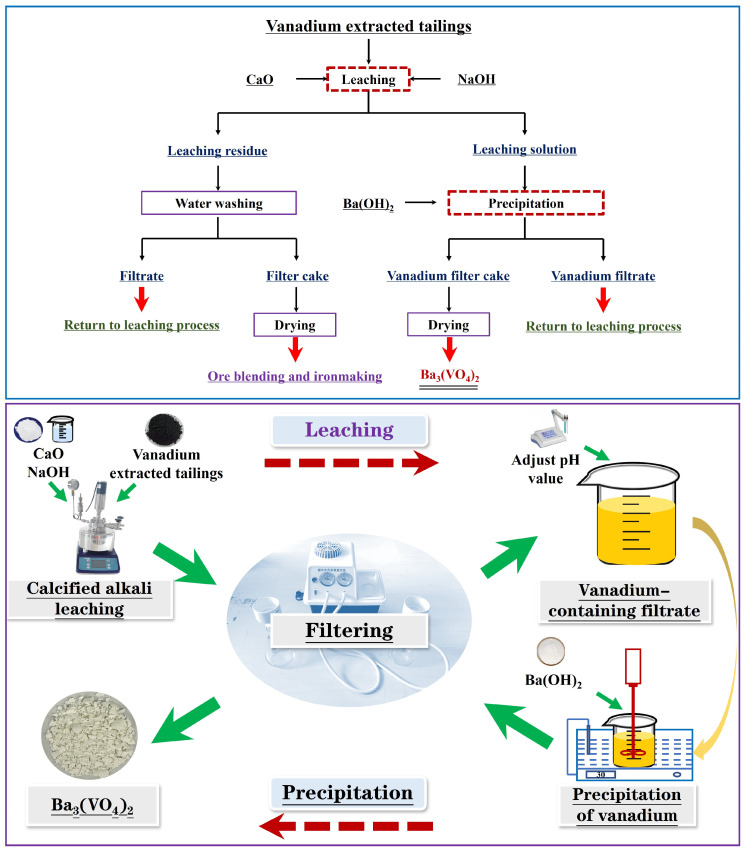
Flow chart for the preparation of barium vanadate from vanadium-extracted tailings.

The process flow and experimental procedure utilized to produce barium orthovanadate from the vanadium-extracted tailings are displayed in Figure 3. First, the tailings were treated with an oxygen-pressure calcification and alkali leaching process. The leached residue was then washed with water to remove any water-soluble sodium ions. This sodium-free residue is suitable for ore reduction in iron smelting. Next, precipitation was performed by adjusting the pH of the leaching solution and adding Ba(OH)_2_ to precipitate the vanadium. The precipitated vanadium product was then dried to produce Ba_3_(VO_4_)_2_. Finally, the wastewater generated by the washing step and the vanadium precipitate mother liquor were mixed with an alkali and recycled back into the leaching process for reuse.

### 2.4. Leaching Mechanism

**Figure 4:**
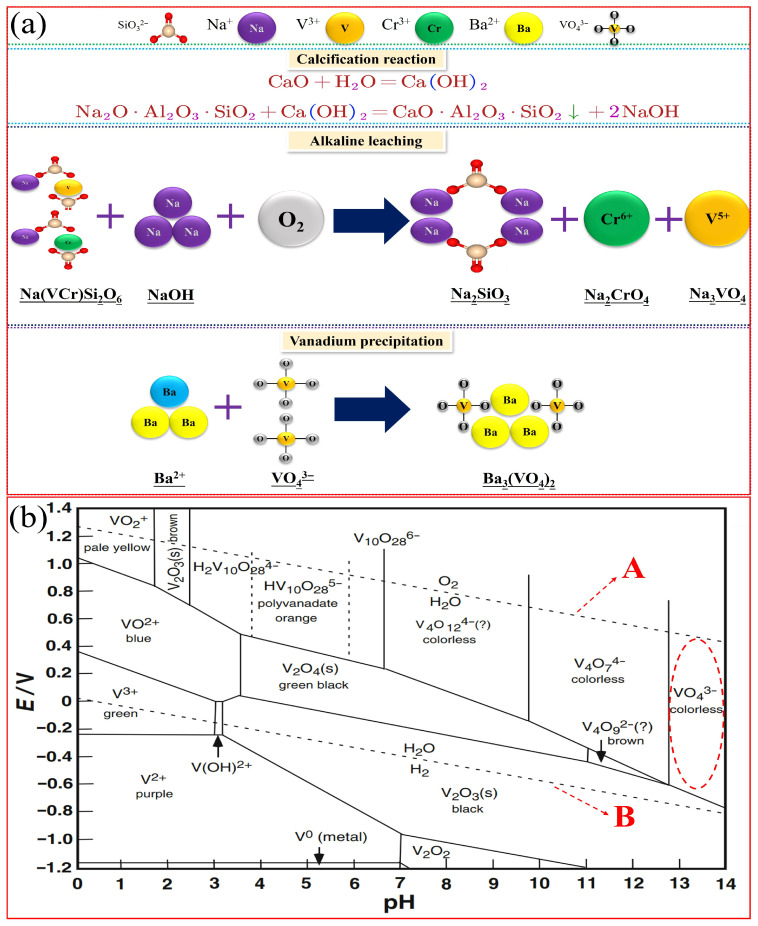
Principles of leaching and precipitation. (**a**) Schematic diagram of the experimental principle; (**b**) E-pH diagram of V-H_2_O system [22] (the question mark indicates uncertainty regarding this substance).

Figure 4a shows a schematic diagram of the calcified alkali leaching process utilized to remove sodium and extract vanadium from the vanadium-extracted tailings. This approach was derived from the lime desodiation process used in the red mud industry [18,19,20,21]. The process was initiated with the hydration of CaO to form Ca(OH)_2_, followed by the dissociation of the Ca^2+^ ions, which diffused into the insoluble sodium-containing phase and replaced the Na^+^ ions through ion exchange. Consequently, the Na^+^ ions diffused into the bulk solution, achieving desodiation. Alkali leaching was used for vanadium extraction because the insoluble vanadium-containing materials in vanadium-extracted tailings mostly consist of sodium vanadiferous pyroxene, which is insoluble in water and dilute acids but soluble in alkali solutions.

### 2.5. Mechanism of Vanadium Precipitation

Using Factsage 8.1, the occurrence, forms, and favorable zones of vanadium in the V-H_2_O system were plotted. As shown in Figure 4b, the E-pH diagram of the V-H_2_O indicates that oxygen evolution reactions occur above line A and hydrogen evolution reactions occur below line B. Thus, the region between lines A and B is the stable zone for water. Within this stable water zone, the solution contains three water-soluble ions, V^3+^, VO_2_^+^, and VO_2_^+^, that can stably exist at pH values below 2. When the pH value exceeds 6 and the solution becomes more alkaline, these ions stably exist as V_4_O_12_^4−^, V_4_O_7_^4−^, and V_4_O_9_^2−^, respectively. Meanwhile, when the pH of the vanadium-containing solution exceeds 13, VO_4_^3−^ becomes the dominant ion. When Ba(OH)_2_ is added, the dissolved Ba^2+^ ions only react with VO_4_^3−^ to form barium orthovanadate, and this process is unaffected by other impurity ions.

### 2.6. Experimental Methods

#### 2.6.1. Calcified Alkali Leaching

**Figure 5:**
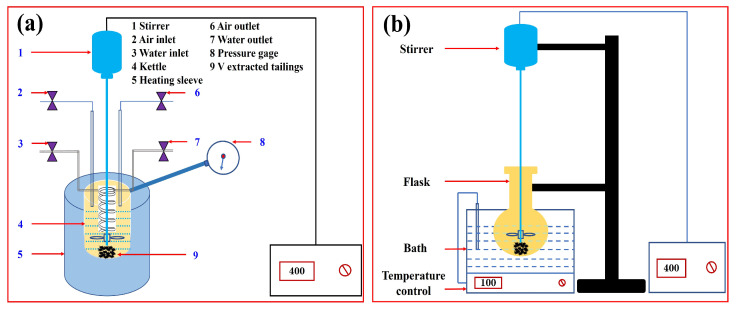
Schematic diagram of the experimental device. (**a**) Pressurized reactor; (**b**) automatic temperature control water bath.

The calcified alkali leaching experiment was conducted in a stainless-steel reactor equipped with heating and stirring devices, as shown in Figure 5a. First, a specific ratio of CaO and vanadium-extracted tailings was added to the reactor, followed by a NaOH solution with a certain concentration. Next, the reactor was secured and completely sealed, oxygen was introduced at a rate of 1 L·min^−1^, and the reactor was heated to a specified temperature. Upon reaching this temperature, the reaction was performed at a stirring speed of 400 rpm for 120 min. After the reaction, circulating cooling water was introduced to reduce the reactor temperature to approximately 50 °C. The reactor was then disassembled, and the reaction slurry was removed and vacuum filtered. The filter cake and filtrate were subsequently collected, and the filter cake was washed with deionized water until a neutral pH was achieved. Finally, the vanadium and sodium content in the final residue were analyzed and the leaching rate was calculated.

The vanadium leaching rate was calculated using Formula (1):

(1) $$\eta_{V}=\frac{M_{1}\times C_{V}-M_{2}\times C_{1}}{M_{1}\times C_{V}}\times 100\%$$

where *η_V_* is the leaching rate of vanadium, %; *M*_1_ is the mass of vanadium-extracted tailings (reactant), g; *C_V_* is the vanadium content in the vanadium-extracted tailings, %; *M*_2_ is the mass of leaching residue, g; *C*_1_ is the vanadium content in the leaching residue, %.

The sodium leaching rate was calculated using Formula (2):

(2) $$\eta_{Na}=\frac{M_{1}\times C_{Na}-M_{2}\times C_{2}}{M_{1}\times C_{Na}}\times 100\%$$

where *η_Na_* is the leaching rate of sodium, %; *M*_1_ is the mass of the vanadium-extracted tailings (reactant), g; *C_Na_* is the sodium content in the vanadium-extracted tailings, %; *M*_2_ is the mass of the leaching residue, g; *C*_2_ is the sodium content in the leaching residue, %.

#### 2.6.2. Vanadium Precipitation

Vanadium precipitation tests were conducted in a round-bottomed flask fixed in a constant-temperature water bath with heating and stirring capabilities. Figure 5b shows a schematic diagram of the apparatus. First, the alkaline leaching solution was collected, and the pH of this leaching solution was adjusted. Based on the vanadium concentration, Ba(OH)_2_ was added in a stoichiometric ratio. The mixture was then stirred and left to react for 30 min. After the reaction, filtration was performed, and the obtained filter cake was washed and dried to obtain the vanadium precipitate.

## 3. Experimental Results and Discussion

### 3.1. Optimization of Calcified Alkali Leaching Process

The main factors affecting the calcified alkali leaching process are the addition of CaO, the alkali concentration, the leaching temperature, and the liquid-to-solid ratio. Therefore, the effects of these factors on the vanadium and sodium leaching rates were evaluated [23].

#### 3.1.1. Effect of CaO Addition Amount

**Figure 6:**
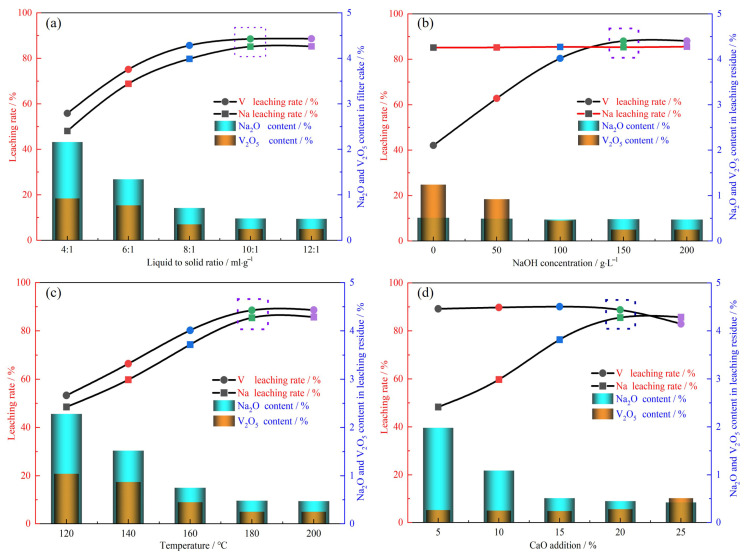
Effect of leaching factors on V leaching rate. (**a**) CaO addition; (**b**) alkali concentration; (**c**) leaching temperature; (**d**) liquid to solid ratio.

The effect of CaO on the leaching rates of vanadium and sodium was investigated by varying the CaO addition amount (5–25% of the weight of the vanadium-extracted tailings). This experiment was performed using 50 g of vanadium-extracted tailings, an alkali concentration of 150 g·L^−1^, a leaching temperature of 180 °C, a liquid-to-solid ratio of 10:1, and a leaching time of 120 min. Figure 6a shows that as the calcium oxide content increases, the vanadium leaching rate gradually decreases. When 15% calcium oxide is added, a vanadium leaching rate of approximately 85% is achieved. Conversely, the sodium leaching rate significantly increases with increasing calcium oxide addition, indicating that sodium leaching is closely related to the presence of calcium oxide and confirming the calcium-replacing-sodium mechanism shown in Figure 4a. The addition of 5% calcium oxide leads to a sodium leaching rate of 44.23%, and increasing the calcium oxide addition amount to 20% improves the sodium leaching rate to 82.36%. Further increasing the calcium oxide content does not significantly change the sodium leaching rate. Taking all factors into account, the addition of 20% calcium oxide optimizes the leaching of both vanadium and sodium.

#### 3.1.2. Effect of Alkali Concentration

The effect of alkali concentration on the leaching rates of vanadium and sodium was investigated within the alkali concentration range of 0–200 g·L^−1^. This experiment was performed using 50 g of vanadium-extracted tailings, 20% CaO addition, a leaching temperature of 180 °C, a liquid-to-solid ratio of 10:1, and a leaching time of 120 min. As displayed in Figure 6b, the sodium leaching rate remains above 82% throughout this experiment, indicating that sodium leaching is unrelated to the alkali concentration. In contrast, the vanadium leaching rate significantly increases with increasing alkali concentration. This indicates that vanadium leaching is closely related to the alkali concentration, confirming the vanadium leaching mechanism shown in Figure 4b. At an alkali concentration of 50 g·L^−1^, the vanadium leaching rate is 62.31%, and at 150 g·L^−1^, the vanadium leaching rate reaches approximately 85%. Further increasing the alkali concentration does not significantly improve the vanadium leaching rate. Therefore, the optimal alkali concentration for achieving the maximum leaching of both vanadium and sodium is 150 g·L^−1^.

#### 3.1.3. Effect of Leaching Temperature

The effect of leaching temperature on the leaching rates of vanadium and sodium was investigated in the range of 120–200 °C. This experiment was performed using 50 g of vanadium tailings, 20% CaO addition, an alkali concentration of 150 g·L^−1^, a liquid–solid ratio of 10:1, and a leaching time of 120 min. Figure 6c shows that as the temperature increases, both leaching rates gradually increase. At 120 °C, leaching rates of about 50% are achieved, and at 180 °C, the leaching rates exceed 80%. Increasing the temperature further does not significantly improve either leaching rate. Therefore, the optimal temperature for leaching both vanadium and sodium is 180 °C.

#### 3.1.4. Effect of Liquid–Solid Ratio

The effect of liquid–solid ratio on the leaching rates of vanadium and sodium was investigated using liquid–solid ratios of 4:1 to 12:1. This experiment was performed with 50 g of vanadium-extracted tailings, 20% CaO addition, an alkali concentration of 150 g·L^−1^, a leaching temperature of 180 °C, and a leaching time of 120 min. As shown in Figure 6d, both leaching rates significantly increase with increasing liquid–solid ratio. Using a liquid–solid ratio of 4:1 provides a vanadium leaching rate of 57.24% and sodium leaching rate of 48.25%. Meanwhile, at a ratio of 10:1, both leaching rates are above 80%. Further increasing the liquid–solid ratio does not significantly change either leaching rate. Therefore, the optimal liquid-to-solid ratio for maximizing the leaching rates of both vanadium and sodium is 10:1 [23].

### 3.2. Vanadium Precipitation

Following the calcified alkali leaching of the vanadium-extracted tailings, the majority of the V and Na entered the leaching solution (see Table 2 for composition). Next, Ba(OH)_2_ was added to the leaching solution to precipitate the vanadium. In this section, the effects of pH value, amount of Ba(OH)_2_, temperature, and precipitation time on the vanadium precipitation rate were studied.

**Table 2:** Content of main components in the leaching solution (g·L^−1^).

Element	V	Cr	Si	P	Mn	Al
Content	0.528	0.004	0.43	0.071	0.022	0.069

#### 3.2.1. Effect of pH

**Figure 7:**
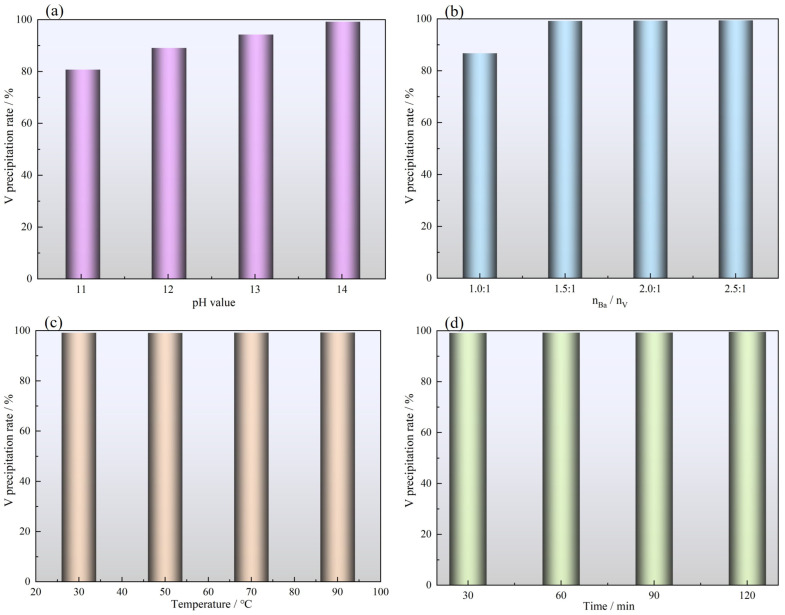
Effect of precipitation factors on V precipitation rate. (**a**) pH value; (**b**) barium hydroxide addition; (**c**) temperature; (**d**) time.

The effect of pH value on the vanadium precipitation rate was evaluated using vanadium-containing leaching solutions with pH values of 11–14. This experiment was performed using 400 mL of leaching solution, 1.03 g of Ba(OH)_2_ ($n_{\mathrm{Ba}}/n_{V}$ = 1.5:1), a stirring speed of 300 rpm, a vanadium precipitation temperature of 30 °C, and a vanadium precipitation time of 60 min. As shown in Figure 7a, the vanadium precipitation rate gradually increases with increasing pH value. When the pH is 14, the vanadium precipitation rate exceeds 99%. Vanadium exists in the form of VO_4_^3−^ at high pH values and reacts with Ba^2+^ to form Ba_3_(VO_4_)_2_ as a precipitate. Therefore, the optimal pH value for vanadium precipitation is 14.

#### 3.2.2. Effect of Ba(OH)_2_ Addition Amount

The effect of Ba(OH)_2_ content on the vanadium precipitation rate was investigated using $n_{\mathrm{Ba}}/n_{V}$ ranging from 1:1 to 2.5:1. This experiment was performed with 400 mL of leaching solution, a pH value of 14, a stirring speed of 300 rpm, a vanadium precipitation temperature of 30 °C, and a vanadium precipitation time of 60 min. As presented in Figure 7b, increasing the amount of Ba(OH)_2_ significantly improves the vanadium precipitation rate. Using $n_{\mathrm{Ba}}/n_{V}$ of 1.0:1 provides a vanadium precipitation rate of just 86.74%. However, when the $n_{\mathrm{Ba}}/n_{V}$ is increased to 1.5:1, the vanadium precipitation rate increases to more than 99%. Further increases in the amount of Ba(OH)_2_ do not affect the vanadium precipitation rate. Therefore, the optimal $n_{\mathrm{Ba}}/n_{V}$ for vanadium precipitation is 1.5:1.

#### 3.2.3. Effect of Vanadium Precipitation Temperature

The effect of the vanadium precipitation temperature on the vanadium precipitation rate was investigated within the range of 30–90 °C. This experiment was performed using 400 mL of leaching solution, 1.03 g of Ba(OH)_2_ ($n_{\mathrm{Ba}}/n_{V}$ = 1.5:1), a pH of 14, a stirring speed of 300 rpm, and a vanadium precipitation time of 60 min. As presented in Figure 7c, the vanadium precipitation rate is unaffected by changes in temperature, with the precipitation rate consistently exceeding 99%. The precipitation of barium vanadate is a rapid process whereby ions combine to form a solid phase, with the bulk precipitation typically occurring instantaneously or within minutes at ambient temperature. Elevating the temperature has a limited effect on enhancing the reaction rate and does not constitute the rate-limiting step in this chemical process. Therefore, 30 °C was selected as the optimal temperature for vanadium precipitation.

#### 3.2.4. Effect of Vanadium Precipitation Time

The effect of vanadium precipitation time on the vanadium precipitation rate was evaluated by performing tests with precipitation times of 20–80 min. This experiment was performed with 400 mL of leaching solution, 1.03 g of Ba(OH)_2_ ($n_{\mathrm{Ba}}/n_{V}$ = 1.5:1), a pH of 14, a vanadium precipitation temperature of 30 °C, and a stirring speed of 300 rpm. As displayed in Figure 7d, the precipitation rate only slightly changes with increasing precipitation time. The precipitation reaction is essentially complete after 30 min of precipitation, with a vanadium precipitation rate exceeding 99%. Therefore, the optimal precipitation time for vanadium precipitation is 30 min.

### 3.3. Characterization of Vanadium Product

**Figure 8:**
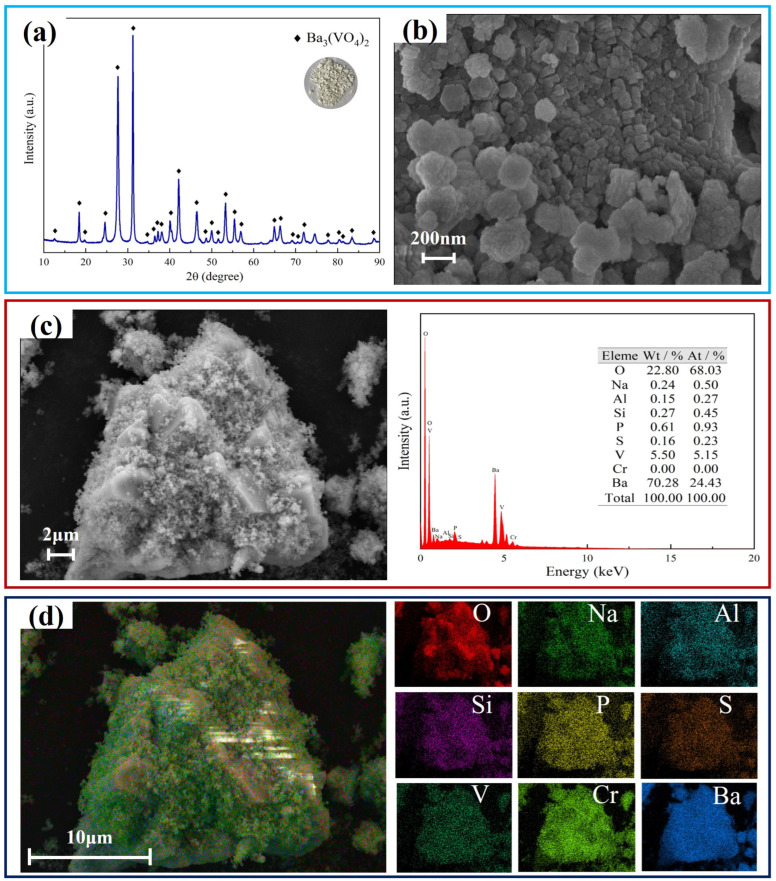
Characterization of vanadium product. (**a**) XRD pattern; (**b**) SEM image; (**c**) EDS results; (**d**) Mappings.

**Figure 9:**
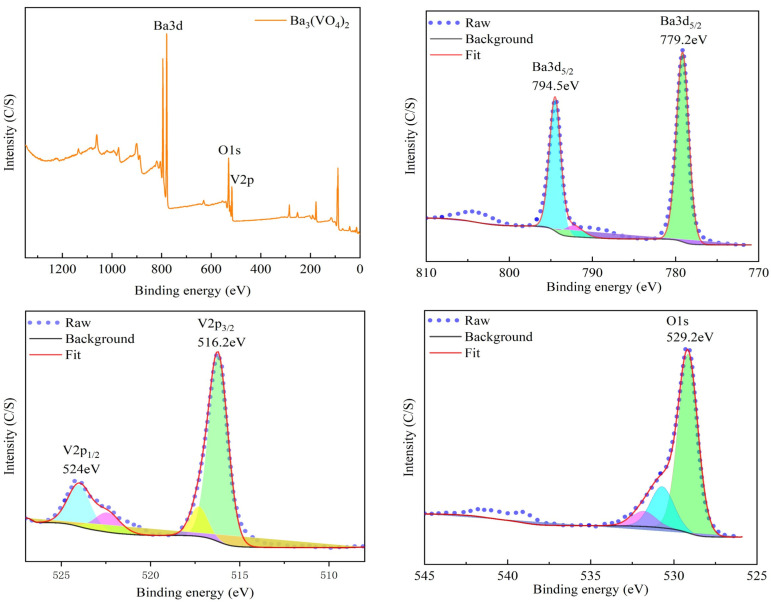
XPS analysis results of vanadium product.

The vanadium product obtained under the optimal precipitation conditions was subjected to three cycles of washing with water, filtration, and drying. Then, the phase composition, microstructure, and composition of the precipitated vanadium product were characterized using XRD, SEM/EDS, XPS, and XRF. As displayed in Figure 8, the precipitated vanadium product consists of a single Ba_3_(VO_4_)_2_ phase, and SEM analysis reveals the small (200 nm) size and amorphous structure of the particles. According to the EDS analysis, the primary constituents of the precipitated vanadium product are barium, vanadium, and oxygen, accompanied by trace sulfur, phosphorus, silicon, and sodium impurities. The elemental mapping images indicate an uneven distribution of O, Na, Al, Si, P, S, V, and Ba within the precipitated vanadium product, suggesting the presence of trace impurities. XPS analysis of the precipitated vanadium product is presented in Figure 9. The full survey spectrum provides a comprehensive representation of the precise elemental composition of the precipitated product, showing distinct peaks at binding energies of 779.2 eV, 794.5 eV, 516.2 eV, 524 eV, and 529.2 eV. These peaks are indicative of the significant bonds associated with Ba, V, and O. Table 3 presents the XRF analysis of the precipitated vanadium product, which is in alignment with the EDS analysis. As indicated by the calculations, the pure Ba_3_(VO_4_)_2_ product has a purity level of 99.52%.

**Table 3:** Main chemical components of vanadium product (mass fraction, %).

Na_2_O	Al_2_O_3_	MgO	SiO_2_	P_2_O_5_	Cl	V_2_O_5_	BaO
0.81	0.37	0.03	0.11	0.12	0.01	24.16	74.4

### 3.4. Characterization of Dealkalized Residue

**Figure 10:**
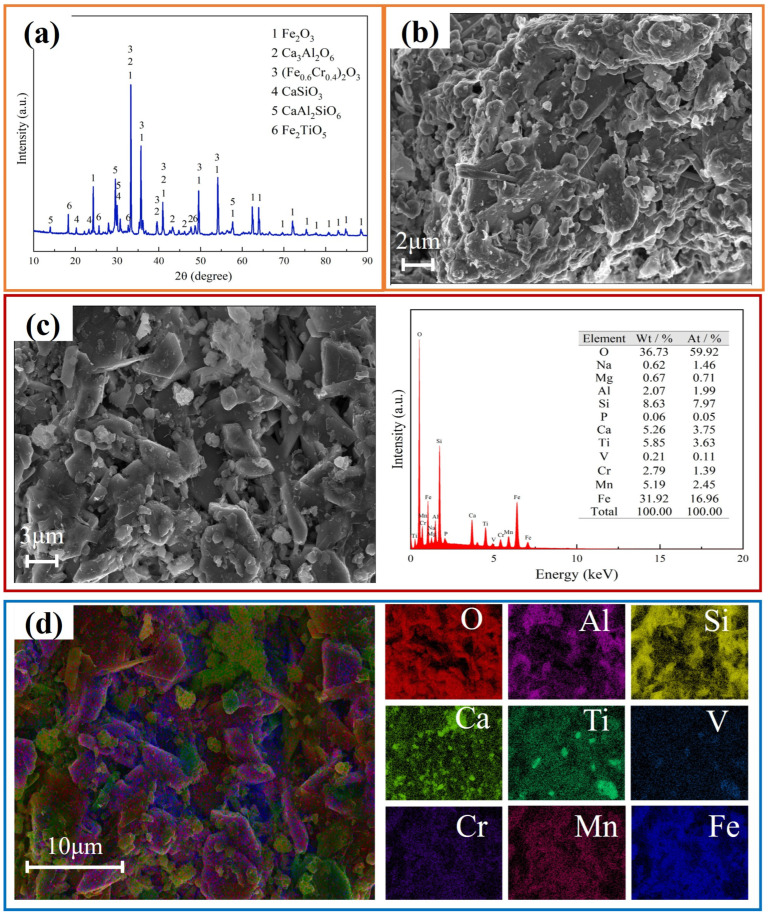
Characterization of dealkalized residue. (**a**) XRD pattern; (**b**) SEM image; (**c**) EDS results; (**d**) Mappings.

The XRD pattern of the dealkalized residue is displayed in Figure 10a. Sodium-containing and vanadium-containing phases are not observed, leaving the calcium-containing phases (Ca_3_Al_2_O_6_, CaSiO_3_, and CaAl_2_SiO_6_) as the main components. This validates the proposed calcified alkali leaching mechanism. The XRF analysis of the dealkalized residue is presented in Table 4. This residue has a lower V_2_O_5_ content of 0.28%, and the Na_2_O content is below 0.5%. These values meet the requirements for ore blending in blast furnace ironmaking (alkali content < 1%). Figure 10b shows an SEM image of the slag after alkaline leaching. The original blocky and rod-shaped structures of the vanadium-extracted tailings are destroyed, and the slag is fragmented and vitrified. The EDS spectrum of this slag is in agreement with the XRF analysis presented in Table 4, showing vanadium and sodium atomic percentages of 0.11% and 1.46%, respectively. Additionally, the elemental mapping images of the dealkalized residue indicate the uneven distribution of multiple elements. The vanadium and sodium projections are relatively dim, indicating low concentrations. Meanwhile, the calcium projection is relatively bright, indicating that the calcium content of the residue is retained after dealkalization.

**Table 4:** Main chemical components of dealkalized residue (mass fraction, %).

Na_2_O	MgO	Al_2_O_3_	SiO_2_	CaO	TiO_2_	V_2_O_5_	Cr_2_O_3_	MnO	Fe_2_O_3_
0.48	2.47	5.16	25.2	21.4	7.03	0.28	2.52	5.46	30.10

## 4. Conclusions

The treatment of vanadium-extracted tailings via calcified alkali leaching enables the leaching of both vanadium and sodium into the leaching solution. The principles of this calcified alkali leaching process and the pH diagram of the V-H_2_O system demonstrate its feasibility. Single-factor experimental studies were performed to determine the optimal leaching conditions, and subsequently, the addition of Ba(OH)_2_ enabled the formation of barium orthovanadate as a product. Then, the components and phases in the dealkalized residue were analyzed, indicating that this residue is suitable for utilization as a blast furnace ore blend for ironmaking. This experimental study may offer valuable insights for the large-scale utilization of vanadium-extracted tailings. The main results are summarized as follows:

(1) Under the optimal leaching conditions (leaching temperature of 180 °C, alkali concentration of 150 g·L^−1^, CaO addition of 20%, and liquid–solid ratio of 10:1), vanadium and sodium leaching rates of 85.25% and 82.36% are, respectively, achieved after 120 min.
(2) In the vanadium precipitation step, a leaching solution pH value of 14 and the optimal addition of Ba(OH)_2_ result in the production of the vanadium orthovanadate product at room temperature within 30 min. Characterization analysis indicates that the product is a single Ba_3_(VO_4_)_2_ phase, and SEM images confirm the small particle size (approximately 200 nm) and amorphous structure of this product.
(3) Analysis of the dealkalized residue shows that the sodium-containing and vanadium-containing phases disappeared after dealkalization, leaving behind the calcium-containing phases (Ca_3_Al_2_O_6_, CaSiO_3_, and CaAl_2_SiO_6_) as the main components. The V_2_O_5_ content of the dealkalized residue decreases to 0.28%, and the Na_2_O content of this residue is less than 0.5%. Therefore, the dealkalized residue fully meets the requirements for blast furnace ironmaking (alkali content < 1%).

## Data Availability

The data presented in this study are available on request from the corresponding author. The data are not publicly available due to privacy or ethical restrictions.
